# The Impact of EBM-Manufactured Ti6Al4V ELI Alloy Surface Modifications on Cytotoxicity toward Eukaryotic Cells and Microbial Biofilm Formation

**DOI:** 10.3390/ma13122822

**Published:** 2020-06-23

**Authors:** Patrycja Szymczyk-Ziółkowska, Viktoria Hoppe, Małgorzata Rusińska, Jolanta Gąsiorek, Grzegorz Ziółkowski, Karolina Dydak, Joanna Czajkowska, Adam Junka

**Affiliations:** 1 Center for Advanced Manufacturing Technologies (CAMT/FPC), Faculty of Mechanical Engineering, University of Science and Technology, Łukasiewicza 5, 50-371 Wrocław, Poland; viktoria.hoppe@pwr.edu.pl (V.H.); malgorzata.rusinska@pwr.edu.pl (M.R.); grzegorz.ziolkowski@pwr.edu.pl (G.Z.); 2 Department of Mechanics, Materials and Biomedical Engineering, Wroclaw University of Science and Technology, 50-370 Wrocław, Poland; jolanta.gasiorek@pwr.edu.pl; 3 Department of Pharmaceutical Microbiology and Parasitology, Wrocław Medical University, Borowska 211A, 50-556 Wrocław, Poland; karolina.dydak@umed.wroc.pl (K.D.); adam.junka@umed.wroc.pl (A.J.); 4 Laboratory of Microbiology, Lukasiewicz Research Network—PORT Polish Center for Technology Development, Stabłowicka 147, 54-066 Wrocław, Poland; joanna.czajkowska@port.org.pl

**Keywords:** additive manufacturing, electron beam melting, surface modification, biofilm, Ti6Al4V ELI, wettability, scanning electron microscopy, computed tomography

## Abstract

Electron beam melting (EBM) is an additive manufacturing technique, which allows forming customized implants that perfectly fit the loss of the anatomical structure of bone. Implantation efficiency depends not only on the implant’s functional or mechanical properties but also on its surface properties, which are of great importance with regard to such biological processes as bone regeneration or microbial contamination. This work presents the impact of surface modifications (mechanical polishing, sandblasting, and acid-polishing) of EBM-produced Ti6Al4V ELI implants on essential biological parameters. These include wettability, cytotoxicity toward fibroblast and osteoblast cell line, and ability to form biofilm by *Staphylococcus aureus, Pseudomonas aeruginosa,* and *Candida albicans*. Obtained results indicated that all prepared surfaces exhibited hydrophilic character and the highest changes of wettability were obtained by chemical modification. All implants displayed no cytotoxicity against osteoblast and fibroblast cell lines regardless of the modification type. In turn, the quantitative microbiological tests and visualization of microbial biofilm by means of electron microscopy showed that type of implant’s modification correlated with the species-specific ability of microbes to form biofilm on it. Thus, the results of the presented study confirm the relationship between such technological aspects as surface modification and biological properties. The provided data are useful with regard to applications of the EBM technology and present a significant step towards personalized, customized implantology practice.

## 1. Introduction

The involvement of new methods of fabrication such as additive manufacturing techniques (AMTs) is undoubtedly required to develop modern, advanced orthopaedic implants. Such methods should allow forming implants from a wide range of materials and to obtain light, durable structures of high biocompatibility and low cytotoxicity. Above-mentioned features are more and more often gained by customized design involving the application of additive manufacturing (AM) technologies. The great advantage of these methods is the high accuracy of manufacturing of finished parts, based on geometric model CAD-3D (computer-aided design). Additive manufacturing techniques allow to form individualized implants, designed on the basis of patient-specific data, obtained from computed tomography. Such implants perfectly mimic the anatomical structure or fill its loss. However, the applicative success of additively manufactured implants strongly depends on their final surface parameters related with the technology applied, manufacturing process parameters, material, and type of post-process used.

One of the representatives of additive manufacturing techniques (AMTs) is electron beam melting (EBM), which belongs to a group of methods called powder bed fusion (PBF). This technology can be successfully used for the production of bone implants from titanium-based alloys. It is based on topical melting and fusion of selective regions of metallic powder particles using high-power electron beam energy [1,2]. The manufacturing process is performed based on customized process parameters in a vacuum chamber at an elevated temperature of around 700 °C, which helps to minimize temperature gradients and local cooling rates. The oxygen-free environment ensures high purity and is suitable for processing materials with high affinity for oxygen, while the elevated temperature minimizes a risk of appearance of residual stresses and, hence, distortion and warpage [3,4]. In these conditions the platform is covered with powder particles forming an even and dense layer, which is subsequently preheated with a high-speed scan of electron beam, followed by selective melting of particles according to 2D cross-section of CAD model. The subsequent layer is built directly on the previous layer, providing durability of the whole model [5]. The process is repeated in a layer-by-layer manner until the desired 3D part is completed.

The EBM process provides advanced products (e.g., scaffolds) of external and internal structure complexity, which is impossible to obtain with traditional manufacturing techniques [6]. Noteworthy, relative material density of EBM-performed structures from Ti6Al4V may even exceed 99.9% [2] and reach mechanical properties comparable to these of bone tissue [6,7]. Combination of low porosity and highly repeatable internal structures allows to design functional properties within the ready product and, therefore, in case of implants, to efficiently mimic replaced bone structures.

To date, numerous studies have been carried out by different research teams to prove the suitability of these processes in implant production [8,9,10,11]. The example of already applied EBM-manufactured implants are replacements of acetabular cups, which have gained approval from United States Food and Drug Administration (FDA) and are CE-certified (Conformité Européenne) since 2007 and 2010, respectively [3].

Nevertheless, there are meaningful issues to be solved if EBM-produced implants are to be accepted for broad use in orthopedics. The optimization of implant surface is one of them, because it should allow bone cells to adhere but at the same time it should impede adhesion of microbial pathogens.

EBM-manufactured implants are characterized by a high roughness. Their surface topography is a consequence of the presence of partially molten and stuck powder particles. Surface topography significantly affects the reactions occurring at the implant–tissue contact zone, ensuring the correct tissue response of cells (e.g., osteoblasts) to the material which promotes cell adhesion [12]. The macroscopic features of the surface determine the type of cells present in the connection area (e.g., there are reports showing that fibroblasts are more likely to occur on smooth surfaces, while osteoblasts are more common in developed areas). In addition, it is worth noting that too high (>2 μm) and too low (<1 μm) roughness (Ra) may have a negative effect on the quality of the connection [13,14]. Therefore, each change in the roughness parameter may have profound consequences on cell growth and proliferation [15,16]. The appropriate surface modification of metal implants may improve compatibility with tissue, affect the rate of bone tissue ingrowth, and reduce the likelihood of infection as well as to increase corrosion resistance, resulting in elevated implantation efficiency [17,18,19]. Thus, the proper selection of modification techniques can be one of the key factors in terms of implant preparation.

On the other hand, high surface roughness may increase a risk of such undesired phenomena as peri-implant infections. These infective diseases, also referred to as the biomaterial-related infections (BRIs), are the second most common cause of implant failure. The reason behind it is surface colonization by microbes and subsequent infection process [20,21], which lead to bone resorption around the implant. When the implant becomes unstable, the transmission of mechanical forces becomes disturbed [22,23]. This results in abnormal bone regeneration or lack of this process. Moreover, if the infection develops from a local into systemic one, it significantly increases the risk of prolonged hospitalization, necessity of re-implantation (or other surgical procedures), and drop of the patient’s life quality [24]. Finally, infection spreading from bone into the patient’s body leads to such significant complications as permanent loss of health or even death [25,26]. Peri-implant infections are initiated by microorganisms’ attachment to the implant and subsequent biofilm formation [27], which, in this particular case, may be defined as a microbial community adhered to a surface, coated with layers of the extracellular matrix. This robust structure protects microorganisms from such unfavorable stress agents as un-optimal temperature, ultraviolet (UV) radiation, drying, or antimicrobial measures [28]. Such therapeutic procedures as surgical debridement or antibiotic therapy (especially the systemic one) display limited effectiveness against biofilm [29,30]. The reason behind this phenomenon is high tolerance of microbes embedded within the biofilm matrix. It is particularly well seen when a comparison between biofilm-forming cells and their planktonic (unbounded, free-swimming) counterparts is performed. It was proven that biofilm displays up to 1000 times higher tolerance to antimicrobials than a planktonic form of the same bacterial strain [31,32]. Moreover, it should be noted that drug penetration through the pathologically altered (hypoxic, necrotic, inflammatory-taken) tissues is significantly reduced [33].

With regard to biofilm formation on additively manufactured implants, our team previously showed that specific type of surface modification strongly correlates with the ability of microorganisms to adhere and develop on it [34,35,36]. Studies have been performed on Ti6Al7Nb scaffolds produced by selective laser melting and subjected to different types of surface modifications, including ultrasonic cleaning and chemical polishing with hydrofluoric and nitric acids [34,36]. The biofilm formation was also verified on different composite mixtures of polylactic acid and hydroxyapatite samples manufactured with additive laser technology [35].

The microorganisms, responsible for the majority of peri-implant infections, belong to so-called opportunistic species, which are often natural residents of the patient’s skin. It is already known that tissues of the peri-implant area display reduced resistance to infection. This zone is called the *locus minoris resistentiae* and it is extremely susceptible to microbial penetration [37,38]. Therefore, orthopedic patients with implanted biomaterials are in a group of high risk of biofilm-related infections [39,40].

It is generally known that the pathological mechanism of peri-implant infection is of complex nature and the main routes of its development depend not only on type of infecting pathogen and the patient’s general health status but also on the properties of the material and surface implant it is built of [17,41,42] (Table 1).

Therefore, the choice of appropriate biomaterial for implant manufacture is of great importance with regard to avoiding possible health complications [27]. Titanium and its alloys are currently considered the most biocompatible of all known metallic materials and, therefore, they are broadly used in the production of many medical devices, including those intended for bone surgery in orthopedics, maxillofacial, and dental surgery. The physical and chemical properties of titanium allow using it safely as material for both short-term and long-term implants (bone stabilizers and dental implants, joint endoprostheses, respectively). Presently it is also known that the most upper surface of a titanium implant consists of titanium dioxide, which greatly improves the ability of the whole structure to permanently connect with live bone tissue [50].

Therefore, the aim of this research was to evaluate the influence of various surface modifications of Ti6Al4V ELI pellets, manufactured with EBM technology, with regard to selected physical parameters of prototypic implants and, in consequence, on their cytotoxicity toward bone-forming cells and the ability of pathogens to form a biofilm on it.

## 2. Materials and Methods

### 2.1. Samples’ Fabrication

**Figure 1:**
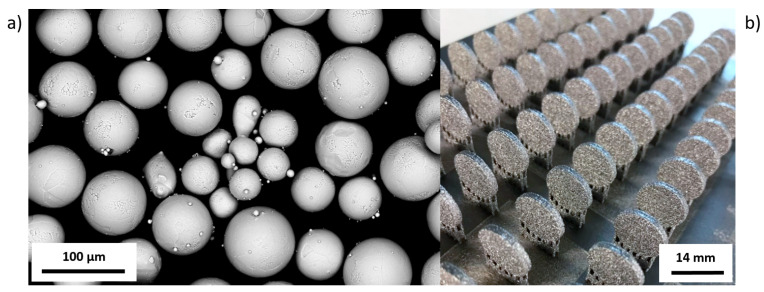
(**a**) SEM image of Ti6Al4V ELI (Arcam AB) powder particles. (**b**) View on the platform with EBM-manufactured pellets.

The specimens in the form of pellets (3 mm thick and 14 mm in diameter) were fabricated with the Arcam A1 device (Arcam AB, Gothenburg, Sweden) with a vertical build orientation (Figure 1). Process parameters were selected in accordance with the range specified by the device manufacturer—beam current: 15 mA, beam spot size: 60 µm, speed: 4530 mm/s, focus offset: 3 mA, line offset: 0.1 mm, layer thickness: 50 µm, pressure in the working chamber: 4 × 10^−3^ mBar. The use of such manufacturing process parameters ensures sufficient material melting and allows achieving high material density (close to 100%).

Samples were made using standard Ti6Al4V ELI Arcam powder in the range of particle size of 45–100 µm. Chemical composition (in wt.%) of the powder used was in accordance with the ASTM F136-13 Standard [51] (Table 2).

In total, 100 samples were produced for testing, which were then subjected to various surface modification processes.

### 2.2. Modifications of Ti6Al4V ELI Pellets’ Surface

To improve the surface quality and to remove powder grains remaining on the surface of the Ti6Al4V ELI samples, three various types of modification were used. The following three types of samples’ surfaces were performed: Group A, as-built samples after EBM process, not subjected to the surface modifications, served as a comparative group; group B, acid-etched samples modified using bath dedicated to titanium alloys in accordance with the guidelines presented in our other paper (briefly, the samples were chemically treated at room temperature for 450 s in ultrasonic bath with the mixture of 5 mL HF (50%) and 15 mL HNO_3_ (50%) in 200 mL of ultrapure water) [36]; group C, sandblasted samples subjected to abrasive blasting with Al_2_0_3_ (p = 30 bar, t = 30 s, distance between nozzle and specimen: 50 mm, angle: 90°); and group D, machined-polished smooth using a grinding and polishing machine (Struers, Ballerup-Copenhagen, Denmark) with the use of liquid colloidal suspension of silicon oxide and polishing cloth.

### 2.3. Surface Topography and Roughness Measurements

Roughness measurements were performed using a laser scanning confocal microscope (CM) Olympus LEXT OLS4000 (Tokyo, Japan). Macro roughness was analyzed with a 20× objective. The Gauss-filtered measurements were set up for an evaluation length of 1.25 mm and a cutoff value of 0.25 mm for machined surfaces, and an evaluation length of 40 mm and a cutoff value of 8 mm for the as-built, acid-etched, and sandblasted surfaces, according to specification standard ISO 4287 [52]. The surface roughness, Ra (the arithmetic mean deviation), was evaluated. Five measurements were performed for each surface.

Areal surface texture data analysis was performed using a computed tomography (CT) device Phoenix v|tome|x (General Electric, Berlin, Germany). All CT measurements were performed at a resolution of 9.14 µm and magnification at the level of 21.86×. The tube voltage and current were kept constant for all scans, namely at 200 kV and 40 mA, respectively, and a copper filter of 0.5 mm was put between X-ray source and sample. Based on CT reconstruction, a qualitative analysis of Ti6Al4V ELI pellets’ surface topography was performed using VG Studio Max 3.0 (Volume Graphics, Heidelberg, Germany), using local advanced surface determination.

### 2.4. Wettability and Surface Free Energy

The wettability of the obtained materials was measured using an OEG SURFTENS UNIVERSAL goniometer (OEG GmbH, Frankfurt, Germany) with integrated SURFTENS software (V 4.7version, OEG GmbH, Frankfurt, Germany) using the sitting drop method, on the basis of which surface free energy was calculated. At least 12 measuring points were made for a given liquid and type of samples. Surface energy calculations were made using the Owens-Wendt-Rabel-Kaelble (OWRK) method [53]. Two liquids of different chemical characterizations were used in the measurement: Water and monoethylene glycol. The dispersion component for water is 21.80 mN/m, the polar component 51.00 mN/m. For monoethylene glycol these components are 29.00 mN/m for the dispersion component and 19.00 mN/m, respectively, for the polar. The water droplet size was 3.9 μL, for ethylene glycol 3.2 μL. Immediately after planting the drops, photos were taken and the shape and volume of the drops were analyzed, the contact angle was measured and the free surface energy of the sample was calculated.

### 2.5. Microbiological Tests

#### 2.5.1. Quantitative Cultures

Applied microbial strains were ATCC6438 *Staphylococcus aureus*, ATCC10231 *Candida albicans,* and ATCC15442 *Pseudomonas aeruginosa.* The procedure of biofilm culturing and quantification was performed analogically to the procedures we presented in our earlier works [34,35,36]. Briefly, strains cultured on the Columbia, Sabouraud, and McConkey agar (Biocorp, Warsaw, Poland), respectively, were transferred to the liquid TSB (Tryptic soy broth) medium and incubated at 37 °C for 24 h in the aerobic conditions. After incubation, density of microbial suspension was measured using a densitometer (Biomerieux, Marcy-l’Étoile, France) and diluted to 3 × 10^8^ cells/mL. Subsequently, EBM samples from groups A, B, and C (Materials and Methods, Section 2.2) were introduced to microbial suspensions for 24 h at 37 °C. After incubation, samples were thoroughly rinsed using physiological saline to remove non-adhered bacteria and to leave only biofilm-forming microorganisms. Subsequently, samples were transferred to the 1 mL of mild detergent (0.5% saponine, Sigma-Aldritch, Saint Louis, MO, USA) and vortexed vigorously for 1 min to free bacterial cells from biofilm extracellular layers. After vortex-mixing, obtained bacterial suspensions were diluted 10–1,000,000 times, then 100 µL of each dilution was cultured on the appropriate stable medium and incubated at 37 °C for 24 h. After this time, microbial colonies were counted and the number of cells forming biofilm on the implants was assessed. All measures were repeated three times. All experiments were performed in triplicate to calculate the average value. Additionally, the numbers of cfu (colony-forming unit) were normalized to the sample surface by the equation: cfu per mm^2^ surface = cfu per sample/surface area mm^2^.

#### 2.5.2. Confirmation of *S. aureus*, *P. aeruginosa*, and *C. albicans* Strains to Form Biofilm on the Tested Biomaterials Using Scanning Electron Microscopy

Results of quantitative microbiological tests were confirmed by visualization of microbial and fibroblasts’ and osteoblasts’ cells using scanning electron microscopy. The microscopy procedure was processed in the following manner: The samples with *S. aureus, P. aeruginosa* and *C. albicans* biofilm on it were fixed using 3% glutarate (POCH, Gliwice, Poland) for 15 min in the room temperature. Thereafter, the samples were rinsed twice with phosphate buffer (Sigma-Aldritch) for the purpose of fixative elimination. The following step was dehydration in increasing concentrations of ethanol (25, 60, 95, 100%) for 5 min in every solution. After rinsing off the ethanol, the samples were dried. Finally, the samples were covered with Au (sputter current: 40 mA, sputter time: 50 s) using QUORUM machine (Laughton, UK) and examined on a scanning electron microscope Zeiss EVO MA25 (Oberkochen, Germany).

#### 2.5.3. Cytotoxicity Assay

A neutral red (NR) cytotoxicity assay was performed in osteoblast (U2-OS, ATCC) and fibroblast (L929, ATCC) cell cultures treated with media used to immerse implants. The procedure was prepared according to ISO 10993 norm: Biological evaluation of medical devices; Part 5: Tests for in vitro cytotoxicity; Part 12: Biological evaluation of medical devices, sample preparation, and reference materials (ISO 10993-5:2009 and ISO/IEC 17025:2005) [54]. Briefly, samples were introduced to the plates’ wells filled with appropriate cell culture media without serum (Sigma-Aldrich) and incubated for 72 h in 5% CO_2_ at 37 °C with shaking at 500 rpm (Schuttler Microplate Shaker, MTS-4, IKA, Königswinter, Germany). After incubation, implants were taken out from the wells, and the plates were spin-centrifuged. The resulting supernatants were next introduced to the osteoblast and fibroblast cell cultures and incubated for 72 h in 5% CO_2_ at 37 °C. After a specific time of incubation, medium was removed and 100 μL of NR solution (40 μg/mL, Sigma-Aldrich) was introduced to each well of the plate. Cells were incubated with NR for 2 h at 37 °C. After incubation, the dye was removed, wells were rinsed with PBS (Phosphate-buffered saline), and left to dry at room temperature. Subsequently, 150 μL of de-stain solution (50% ethanol, 49% deionized water, 1% glacial acetic acid (v/v); POCH) was introduced to each well. The plates were vigorously shaken in a microtiter plate shaker for 30 min until NR was extracted from the cells. Next, the value of NR absorbance was measured spectrometrically using a microplate reader (Multi-scan GO, Thermo Fisher Scientific, Waltham, MA, USA) at 540-nm wavelength. The absorbance value of cells not treated with extracts was considered 100% of potential cellular growth (positive control sample).

#### 2.5.4. Statistical Analysis

Statistical calculations were performed using Statistica software (ver 10, StatSoft Inc., Kraków, Poland) and the Mann-Whitney (statistical significance was defined as *p* < 0.05) or Kruskal-Wallis test with post hoc Dunn’s analysis (*p* < 0.001).

## 3. Results

### 3.1. Samples’ Fabrication

Pellets were made of titanium alloy (Ti6Al4V ELI) powder in the range of particle size from 45 to 100 μm (Figure 1a). The results of the building of pellets produced using EBM are shown in Figure 1b.

**Figure 2:**
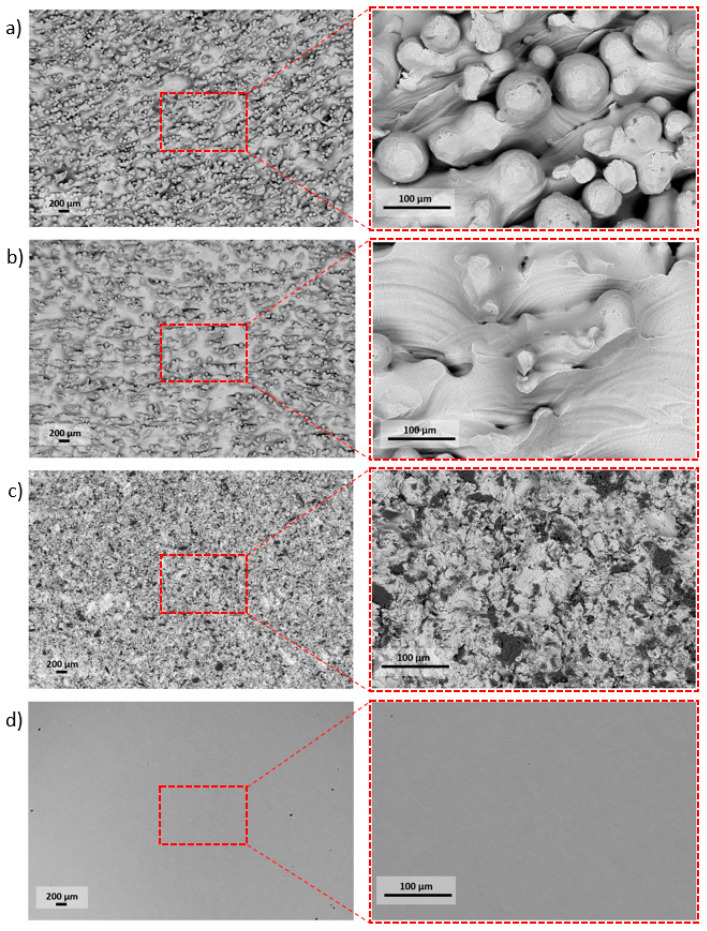
The high diversity of pellets’ surfaces followed by the specific type of surface treatment: (**a**) As-built, (**b**) acid-etched, (**c**) sandblasted, (**d**) machined.

Samples for further testing were prepared by removing supports and cleaning the surface using isopropanol bath. Subsequently, pellets were subjected to the surface modifications, as it was described in Materials and Methods, Section 2.2. Electron microscope micrographs of samples of four above-mentioned groups are presented in the Figure 2.

**Figure 3:**
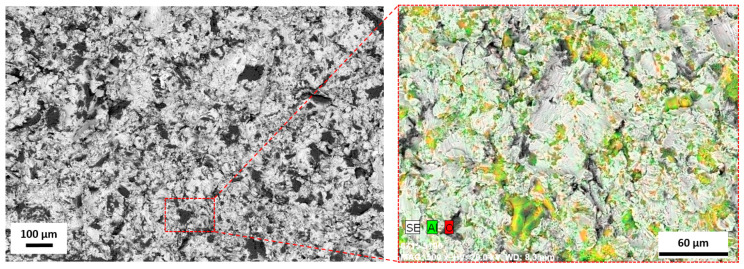
The element distribution map for a sandblasted surface. The colored surface indicates area with Al_2_O_3_ particles SEM/EDS.

The research carried out using SEM was used to determine the surface morphology. Surfaces after the modification methods used were characterized by high diversity. On the surface of the as-built specimen (Figure 2a), spherical particles of material were visible, which were part of the nonmelted powder on the surface, being an artifact after the EBM process. After chemical etching (Figure 2b), a change in the surface was noticeable—no spherical powder particles were present, while smoothed surfaces and a visible dissolution of some narrow edges occurred. The surface after sandblasting (Figure 2c) was characterized by a large surface development, due to the presence of a high number of craters and embedded abrasive particles on the surface (confirmed by an EDS analysis—Figure 3). Surface after machining was smooth, and only thin lines after polishing were visible (Figure 2d).

### 3.2. Surface Topography and Roughness Measurements

**Figure 4:**
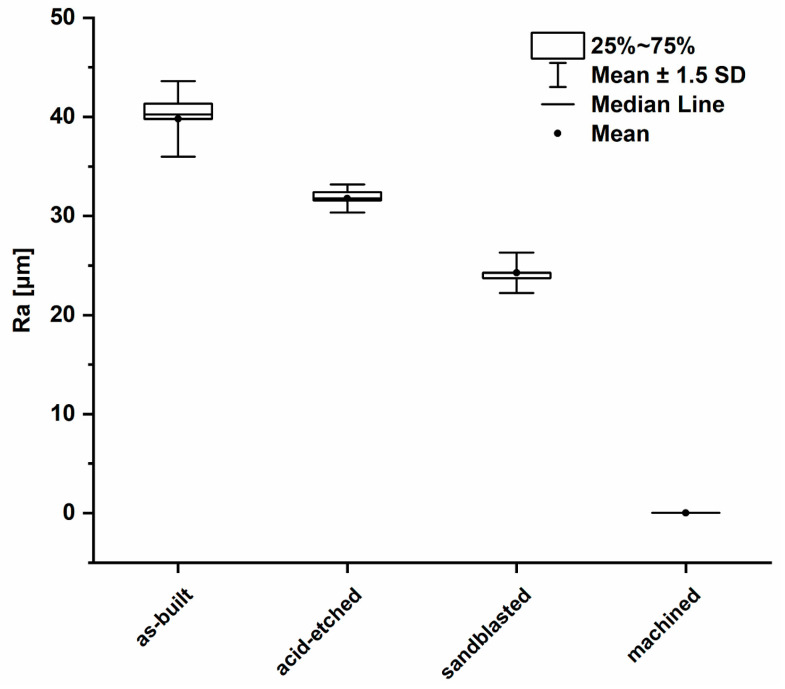
Box plot of the average surface roughness values (average values and standard deviation).

**Figure 5:**
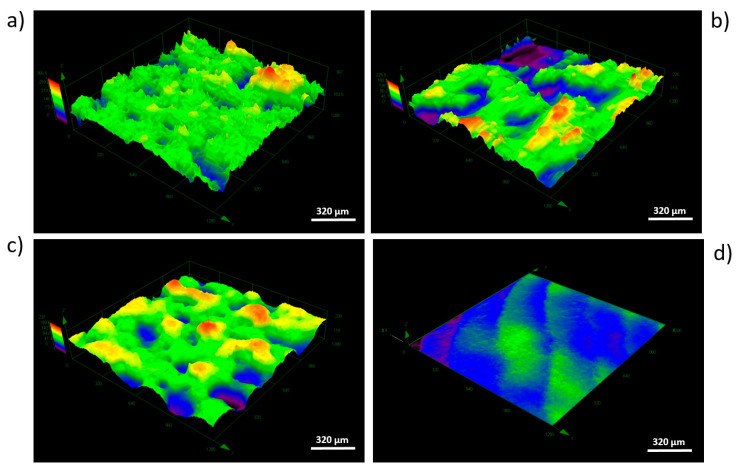
Overview of 3D surface maps for selected area (dimetric projection) obtained by four types of surface modifications: (**a**) As-built, (**b**) acid-etched, (**c**) sandblasted, (**d**) machined (area of 1280 μm × 1280 μm), CM.

The average values of surface roughness (Ra) obtained, as in a result of confocal microscopic (CM) analysis, are shown in Figure 4. The Figure 5 shows 3D surface maps obtained depending on the applied modification method. The highest value of Ra parameter (av. 39.79 ± 2.54 µm) was observed for as-built parts, due to the occurrence of nonmelted parts of powder. The surface differentiation was additionally confirmed by the occurrence of the highest dispersion visualized, as evidenced by the long whiskers in box plot of Figure 4. In the case of surfaces after modifications, a noticeable decrease of the Ra parameter was (observed. For acid-etched surfaces, the average Ra was 31.77 ± 0.94 µm and for sandblasted surfaces the average Ra was 24.27 ± 1.36 µm. Machined surfaces had a lower dispersion of roughness results than as-built surfaces, which manifested as a reduced size of the box and shorter whiskers. The lowest value of Ra was observed for machined surface (Ra average value = 0.033 ± 0.003 µm) and the average line coinciding with the median on the box plot.

**Figure 6:**
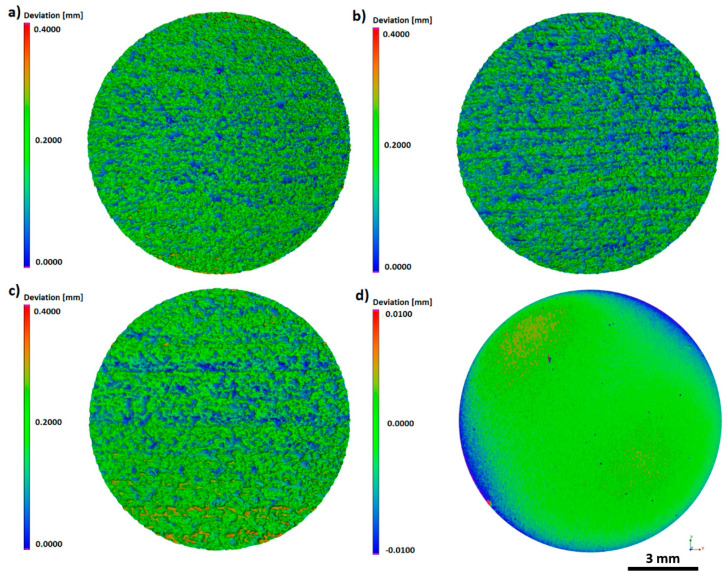
The 3D topography of the entire sample surface: (**a**) As-built, (**b**) acid-etched, (**c**) sandblasted, (**d**) machined, CT.

The use of computed tomography allowed obtaining three-dimensional areal surface topography images for the entire sample geometry (Figure 6). As shown by CT, the additively manufactured titanium pellets were characterized by a directional surface texture caused by the layer-by-layer process. Areas with increased irregularity, associated with the occurrence of nonmelted powder particles, were also identified.

The same trends were observed during examination of texture area data. The as-built surface was characterized by an irregular surface consisting of peaks and valleys (Figure 6a). Hence, the developed surface area ratio was the highest and equaled 218.0133 mm^2^. On acid-etched surfaces, the smoothness of pointed hills and the presence of the increased number of flat depressed areas were observed (Figure 6b). The used chemical treatment method reduced the surface expansion to the level of 189.9642 mm^2^. The surface after sandblasting displayed a different morphology, which was caused by the abrasive particles remaining on the surface, confirmed by microscopic analyses. The depth decreased significantly as a result of this modification process and this also caused a lowering in the surface area ratio to the value of 181.5815 mm^2^ (Figure 6c). The surface after machining was smooth with no elevations or depressions, with a surface expansion parameter of 113.5366 mm^2^. Occurring surface irregularity, especially on the edges of the sample, was associated with the polishing procedure used, which included a semi-automated process.

Requirements for the adequate characterization of roughness depend greatly on the specific application. It should be noted that roughness analysis carried out on selected line profiles might not properly reflect the three-dimensional surface topography, especially for elements manufactured additively, for which the resulting surface topography was related to the applied process parameters and manufacturing conditions (e.g., building orientation, scanning strategy).

### 3.3. Surface Free Energy and Wettability

**Figure 7:**
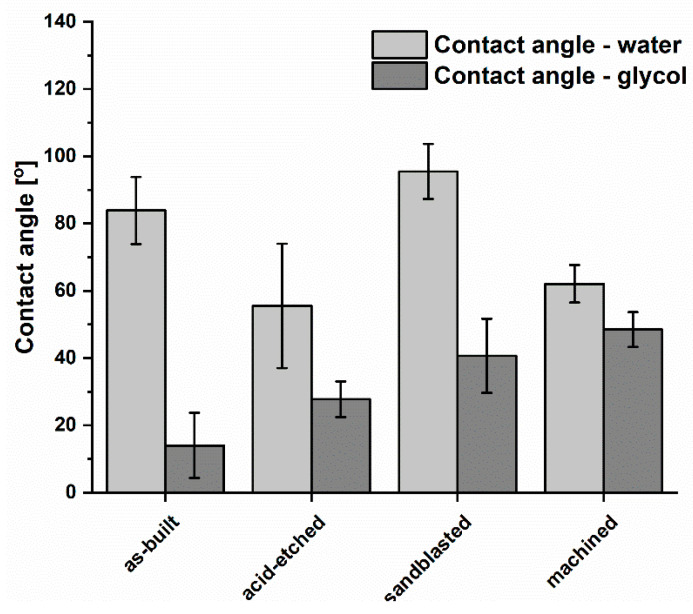
Comparison of wettability of titanium alloy surface after various types of treatment (average values and standard deviation).

Results of wettability test with regard to analyzed structures is presented in Figure 7. The as-built surfaces displayed a hydrophilic effect (the contact angle with water was calculated for 83.9° ± 10°). The machined and acid-etched surfaces were of hydrophilic properties (contact angle < 90°). In turn, only samples treated with sandblasting were characterized by a hydrophobic surface with a water contact angle of more than 90°. Such an effect may be a result of surface’s significant roughness and the expansion of its microstructure, which significantly affected the philic-phobic nature of the modified surface [55]. Changes in the nature of surface topography caused the formation of air pockets on extensive, rough surfaces. It had a significant impact on the intermolecular interactions between the liquid and solid phase (liquid/metallic surface) and increased the value of the contact angle. In the case of contact angles with monoethylene glycol, the highest angles were obtained for sandblasted samples (40.7° ± 11.0°). For acid-etched surfaces, these values were 27.8° ± 5.3°. The lowest values of wetting with monoethylene glycol were obtained for the as-built surface, respectively 14.00° ± 9.70°.

**Figure 8:**
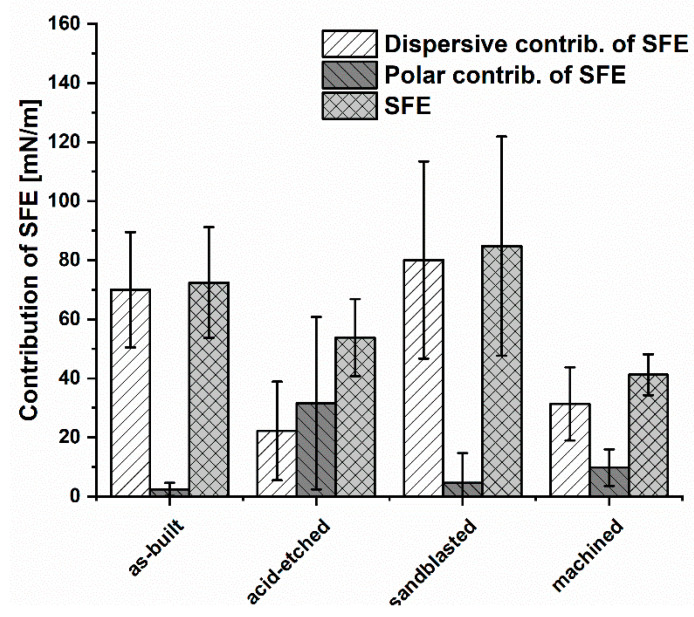
The contribution of polar and dispersion components in SFE and SFE values for the analyzed materials (average values and standard deviation).

The lowest SFE (surface free energy) for the analyzed materials (Figure 8) was obtained for machined samples (41.2 ± 6.9 mN/m). Samples subjected to the sandblasting process had the highest SFE values of 84.8 ± 37.1 mN/m and were similar for the values obtained for as-built surfaces, 72.4 ± 18.8 mN/m.

Considering the share of dispersion and polar components in the total SFE of the analyzed materials, it could be seen that the dispersion component of as-built, sandblasted, and machined surfaces contributed to SFE in the highest manner. Therefore, the dominant contribution in SFE of these samples was occupied by dispersion interactions, i.e., London forces. In turn, on surfaces subjected to acid-etching, the polar component had a greater proportion of total SFE. This showed that such polar interactions as hydrogen or dipole-dipole bonds, which can be the result of an increased number of hydroxyl groups on the surface, have a significant impact on wettability. It confirmed that the chemical treatment significantly modified the surfaces in physicochemical terms. In this case, polar interactions, i.e., acid-base, hydrogen bonding and others, have a greater contribution in SFE compared to other analyzed samples.

### 3.4. Biological Tests

**Figure 9:**
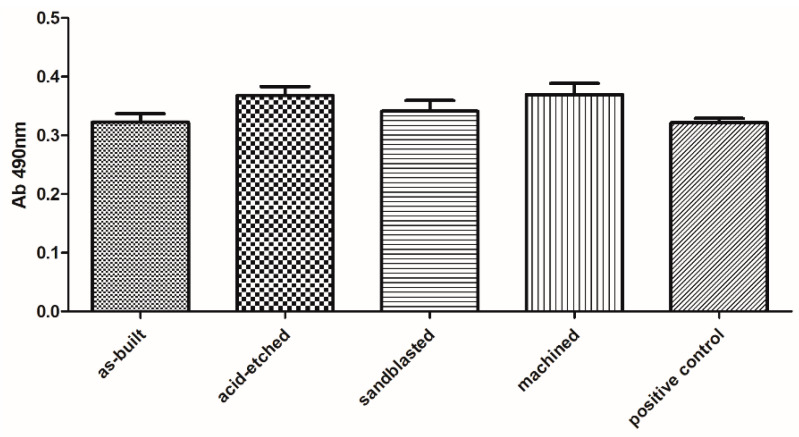
Survival of osteoblasts in media, in which surface-modified samples were immersed, compared to untreated osteoblasts (positive control box) (average values and standard deviation). No statistically significant differences were observed between particular groups (K-W test, *p* > 0.001).

Biological analyses revealed that samples manufactured using EBM technology displayed no cytotoxicity toward osteoblasts, regardless the surface treatment. The observed higher survival of osteoblasts in medium where samples were incubated comparing to positive control samples was statistically insignificant (K-W test, *p* > 0.05) (Figure 9).

**Figure 10:**
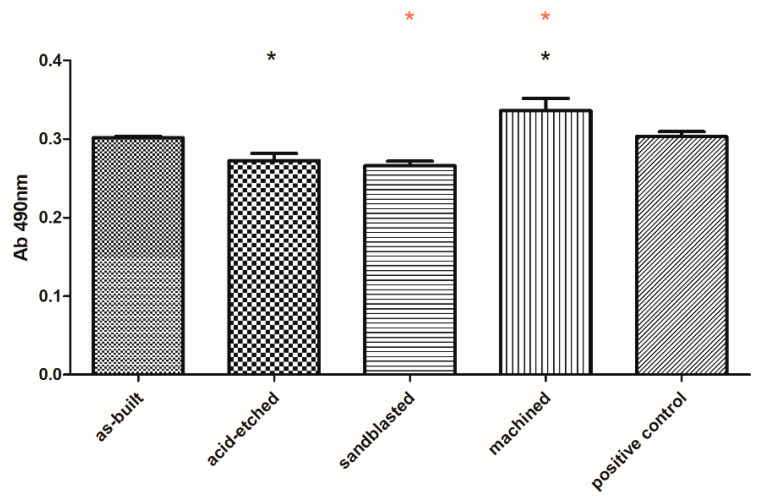
Survival of fibroblasts in media, in which surface-modified samples were immersed, compared to untreated fibroblasts (positive control box) (average values and standard deviation). Asterisks mark statistically significant differences between specific types of samples (K-W test, *p* < 0.001).

The lack of significant toxicity of the investigated materials was confirmed also for fibroblast cells. But, in this case, a clear difference between the analyzed series was observed. Machined samples were less cytotoxic than sandblasted and acid-etched samples, while their cytotoxicity (at very low level) was comparable to cytotoxicity of as-built samples (Figure 10).

**Figure 11:**
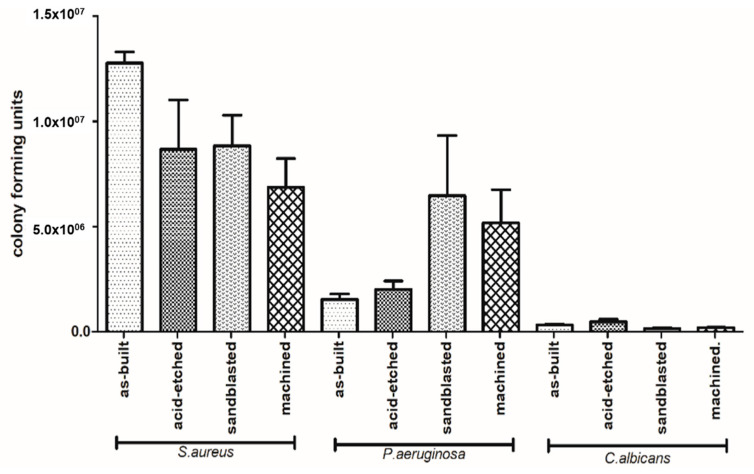
Differences in number of microbial cells forming biofilm on particular types of samples (average values and standard deviation).

The species-specific differences in ability to form biofilm on analyzed surfaces is presented in Figure 11. Regardless of sample type, *C. albicans* cells formed the weakest and *P. aeruginosa* formed, the average, while *S. aureus* formed the strongest biofilm structure in terms of number of microbial cells. This difference was statistically significant (Kruskal-Wallis test with Dunnett’s post hoc analysis, *p* < 0.001).

The number of biofilm-forming cells adhered to the implants depended on the type of surface modification (Figure 11). In the case of *C. albicans*, the highest number of biofilm-forming cells was detected on acid-etched samples, next on the as-built samples, and then on the machined samples, while the lowest number of *C. albicans* cells was detected on sandblasted samples. The highest number of *P. aeruginosa* biofilm-forming cells was detected on sandblasted samples, next on machined samples, followed by acid-etched and as-built samples. *Staphylococcus aureus* formed the biofilm the most eagerly on as-built samples, followed by acid-etched, sandblasted, and machined samples, finally.

Analysis of differences in ability to form biofilm by investigated species on the same type of sample revealed that *S. aureus* formed significantly stronger biofilm on as-built surfaces than *P. aeruginosa* and *C. albicans*; the same pattern was observed when machined and acid-etched samples were used as a surface for biofilm formation. In the case of sandblasted samples, not only *S. aureus* but also *P. aeruginosa* formed significantly stronger biofilm than this one of *C. albicans* (K-W test, post hoc Dunnett’s analysis, *p* < 0.001).

**Figure 12:**
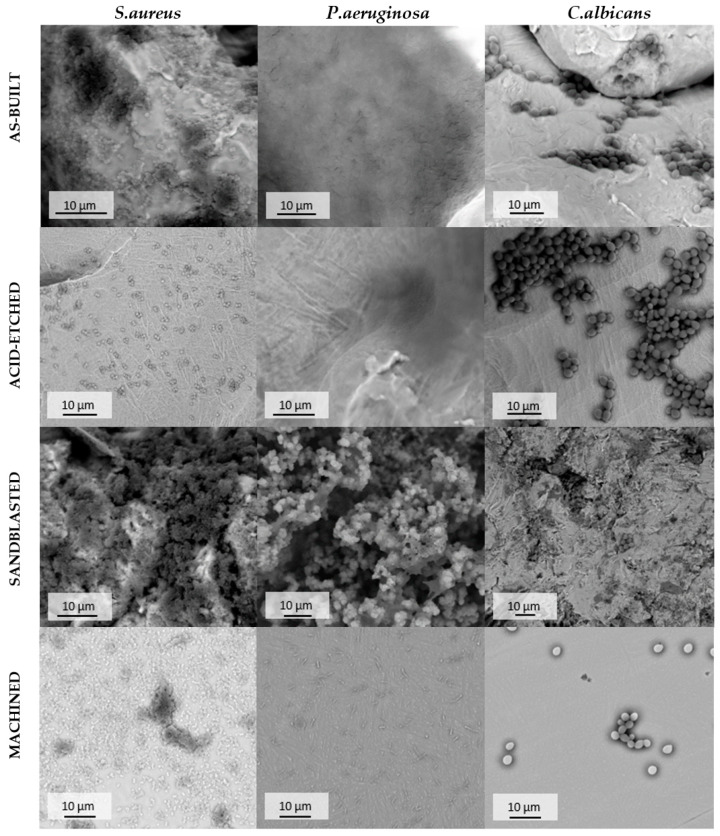
Adhered cells of *S. aureus*, *P. aeruginosa* and *C. albicans* on the surface of additively manufactured samples subjected to various modification processes, SEM. For quantitative results of biofilm-forming cells of particular pathogens on tested types of pellets refer to Figure 11.

SEM analysis confirmed the above-presented quantitative results. As it can be seen in Figure 12, surfaces of samples after EBM process and after corundum blasting were covered with a dense layer of *S. aureus* cells. However, after chemical polishing, bacterial cells were less numerous and formed small clusters instead of the previously mentioned dense cell layer. A similar phenomenon was observed with regard to mechanically polished samples where microbiologically intact surfaces of the biomaterial were visible. The SEM visualization revealed the analogical pattern in the case of *P. aeruginosa* cells. In turn, regardless of the surface type, the *C. albicans* cells were observed on all tested surfaces. However, they did not form the robust biofilm but relevantly low-numbered cellular clusters. Therefore, results obtained using the SEM method stayed in line with data presented in Figure 12.

## 4. Discussion

The use of biomaterials for bone implantation is one of the key risk factors of infections referred to as biomaterial-related infections (BRIs). Once settled by biofilm-forming microorganisms, implants provide them a surface to spread to the neighboring tissues and, finally, throughout the whole patient’s body [56]. One of the main aims of the material science branch dealing with bone implants is to provide surfaces that would be nontoxic to bone-forming cells and, at the same time, would impede adhesion of biofilm-forming microorganisms. To date, a number of studies aiming to provide structure of biomaterials resistant to microbial colonization but appropriate for colonization by bone-forming cells was performed [57,58,59]. Despite the above-mentioned, ongoing efforts of the scientific environment, the relationship between surface roughness and cell adhesion still remains largely unelucidated. Therefore, the aim of the present study was to evaluate the impact of EBM-manufactured Ti6Al4V ELI alloy surface modifications on cytotoxicity toward eukaryotic cells and microbial biofilm formation.

In the first step of research, using Ti6Al4V ELI powder (Figure 1) and subsequent modifications (sandblasting, chemical etching, machining), we manufactured biomaterials of a highly diversified surface (Figure 2 and Figure 3).

The typical purpose of such modifications is to create the implant surface irregularities suitable for the adhesion of eukaryotic cells and, on the other hand, to prevent colonization of bacteria on the surface of the implant. The surface of titanium alloys plays a key role in implant integration in the human body. Therefore, it is possible to affect cell adhesion by producing specific surface topography using, e.g., mechanical methods such as machining or sandblasting [60]. We decided to perform sandblasting, which provides a predictable attachment and growth of soft tissues (e.g., periodontal ligament fibroblasts) on the modified titanium surface [61]. Sandblasting and machining causes the roughness peaks derived from nonmelted powder particles to be abraded and, therefore, the entire surface was smoothed compared to as-built samples. Chemical methods such as acid treatment are used to remove the oxide layer and contamination in order to obtain clean and uniform surface finishes. Specific surface topographic changes are leading to improve the adhesion [61]. Unfortunately, the chemical etching is not adapted to the finishing of the parts produced by EBM directly at the exit of the process. Other finishing operations for the functional surfaces remain necessary [62]. In earlier studies, it was shown that surface roughness of titanium alloy in the range of 0.5–1.5 μm leads to stronger anchoring of hard tissues than a surface that is smoother or rougher than above-mentioned range [63,64,65].

Out of the four types of surfaces we obtained, the smoothest were the machined samples, next were the sandblasted samples, followed by the acid-etched samples, and the lowest smoothness was displayed by the as-built samples (Figure 4). Data summarized in Figure 4 are of great importance with regard to the matter analyzed, because numerous research indicates the significant reduction of biofilm growth on polished surfaces [66,67,68,69], specifically when a threshold surface roughness value did not exceed 0.2 μm [70].

In the case of samples produced in this research, a significant reduction in roughness was obtained for sandblasted samples (Ra = 24.27 ± 1.36 µm), whereas only machined ones showed a comparable threshold value, with an average of Ra = 0.033 ± 0.003 µm (Figure 4). Systematic reviews conducted by Teughels et al. [71] and Truong et al. [72] indicate that surface roughness had a predominant effect over hydrophilicity [72] and that other interfacial parameters had little or no relevance [72]. From the other hand, it needs to be emphasized that there are reports indicating that not only microbes, but also bone-forming cells, prefer a porous and uneven surface over these polished ones [73,74,75]. In light of the above-mentioned data, samples manufactured in this research presenting higher Ra than polished ones, might be of higher potential applicability within the patient’s body then these of lower Ra.

As it can be in Figure 5 and Figure 6, the as-built, acid-etched, and sandblasted samples were covered with craters of approximate diameters of a few hundred micrometers; such valleculas would be sufficient to accommodate both microbial multicellular aggregates (for example, single staphylococcal cell is of 1-micrometer diameter) or a few eukaryotic cells at the same time (for example, osteoblasts are of approximately 50-micrometer diameter) [76]. Therefore, it seems to be reasonable that the developed area ratio should also be included in microbiological context, as the substratum contact area available to cells plays a key role in governing their adhesion and also contributes to the achieved hydrophobicity of the surface [77]. The adhesion potential between the cells and the biomaterial surface is an extremely complex phenomenon and depends on such factors as the physicochemical properties of the cells themselves and the physicochemical properties of the substrate as well as the environmental conditions in which the attachment occurs [27,78].

Research concerning the optimal topography for the bone and soft tissue contact remains currently unanswered. In addition to surface topography, there is a possible relationship between the role of wetting properties for the interfacial biological responses and the interrelating effects of topography and wetting. Therefore, we conducted research on the wettability and contribution of polar and dispersion components in SFE values for the analyzed surface-modified materials (Figure 7 and Figure 8, respectively). In the case of obtained results on wettability, it may be stated that only sandblasted surfaces may be weaker wetted with water than machined, acid-etched, and as-built samples. Surface composition and hydrophilicity are parameters that play an important role in the implant–tissue interaction and osseointegration because highly hydrophilic surfaces seem more desirable in view of their interactions with biological fluids, cells, and tissues [79,80]. Moreover, surface free energy is a dominant factor from a high number of parameters conditioning cell adhesion and proliferation, but roughness can strongly disturb the relationships between surface free energy and cell proliferation [16].

In turn, analyzing the obtained results concerning SFE values (Figure 8), it may be presumed that acid etching and machining samples, which displayed the lowest aforementioned parameters among tested specimen, would be less wetted by the given media and, thus, they would provide better protection against corrosion [81,82]. From the other side, one should always remember that body fluids are always water-based. Therefore, the above-mentioned surfaces may not show the desired biological properties (biocompatibility) in human organism, and do not integrate sufficiently with bone tissue of host. Analysis of the values of polar and dispersion components as well as SFE (Figure 8), revealed that surface modification treatments by mechanical factors (wrapping, grinding) do not change the overall trend of the share of individual components in the total SFE (in this case, the dispersive components dominated). One should, however, notice that chemical treatment changed the home component in SFE to a polar one (Figure 8), which suggests that the surface was modified not only physically (change in roughness) but also chemically. It stayed in line with results provided by Elias et al., who stated that sandblasted and acid-etched implants, due to the homogeneous surface roughness and porosity, allow for better cell adhesion than in implants without surface treatment [16]. The cytotoxicity assays performed on the osteoblast cell line in this research indicated that any of the applied surface modification had a statistically significant impact on these cells’ survival (Figure 9). In turn, our results indicated that sandblasted and acid-etched specimens decreased survival of fibroblasts in a statistically significant (K-W test, *p* < 0.001) manner in comparison to machined samples (Figure 10). No statistically significant difference in cytotoxicity level in fibroblasts was observed when they were exposed to as-built vs. machined specimen. The explanation of the above-mentioned differences remains, at the moment, hypothetical and requires further experiments. However, one may assume that osteoblasts, thanks to their ability to produce a high concentration of alkaline phosphatase, may be able to neutralize acidic remnants of the acid-edged surfaces [76]. The presence of the remaining, such as $O_{2}^{-}$, $O_{2}^{2-}$, and OH^−^, ions that display antimicrobial activity on the surfaces after chemical etching was reported in other studies of ours and by other research teams [34,36,83]. We are aware of the fact that another necessary step to take is testing of osteoblast in in vitro colonization and we plan to undertake it in our next line of investigation, as a stage preceding animal model studies. Nevertheless, the aforementioned favorable results showing lack of cytotoxicity of EBM-produced, surface-modified implants, performed by the normative method presented in this research, indicated the righteousness of this research direction.

According to data presented in Figure 11, staphylococcal biofilm grew the most eagerly on the as-built surfaces. The *P. aeruginosa* grew in the most robust manner on the sandblasted specimen, while the *C. albicans* developed the strongest biofilm on acid-etched samples. The *C. albicans* is known for its ability to thrive in acidic conditions (including stomach environment [84]), while the optimal pH conditions for *P. aeruginosa* are between 6.6–7.0 [85]. It may explain the reason for weak development of pseudomonal biofilm on acid-etched surfaces and the strong development of *C. albicans* on the same type of sample (Figure 11). The *S. aureus* is, in turn, the most spread nosocomial pathogen, mostly due to high ability to form biofilm on virtually all types of surfaces [86,87]. Indeed, data presented in Figure 11 show a lack of significant correlation between the type of modified surface and ability of *Staphylococcus* to form biofilm on it. It should be also noticed that relatively high standard deviations observed in a number of cells of the same pathogen within technical repeats are well-known and still unsolved phenomenon in biofilm studies [88] and they are a result of a plethora of factors related with type of cells, biofilm matrix, medium, and surface of growth. Nevertheless, results presented in Figure 11 and additionally backed up by SEM imaging (Figure 12) indicate the sandblasted surfaces are the least usable as future implants in the patient with the medical history of staphylococcal infections.

The biological results presented in this study, supported with data on implant mechanics and local epidemiology, show that all EBM-manufactured Ti6Al4V ELI alloy surface modifications are potentially applicable for bone implant development with regard to low cytotoxicity toward eukaryotic cells. The next line of investigation we are planning to perform on animal model should provide conclusive results necessary to launch studies on human patients. The very crucial data provided here with context to matter analyzed concern the differentiated ability of specific pathogens to form biofilm on Ti6Al4V ELI alloy surfaces subjected to the specific types of modification. Implementation of this knowledge with data on patient’s etiological factors of infection may lead to a choice of implant whose surface would impede biofilm development and allow bone-forming cells to develop in an undisturbed manner. Such an approach may be another step in the direction of highly customized, personalized implantology.

## 5. Conclusions

(1) The presented surface modifications of the EBM-processed Ti6Al4V ELI alloy changed their surface topology, wettability, and SFE parameters.
(2) The lack of toxicity of all investigated materials was confirmed for osteoblasts. The acid-etching and sandblasting modifications decreased viability of fibroblasts in cytotoxic tests. No cytotoxicity was displayed for fibroblasts when machined samples were applied.
(3) The microbiological tests indicated that the as-built, the sandblasted, and the acid-etched alloy should be not applied in patients with a history of infection caused by *S. aureus*, *P. aeruginosa*, or *C. albicans*, respectively. In such a case, the machined, as-built, and sandblasted alloys should be applied, respectively.
(4) In patients with an unknown history of microbiological infection, the Ti6Al4V ELI alloy of the machined surface should be applied, because of the lowest cytotoxicity combined with the most favorable joined results concerning the ability of biofilm formation by analyzed pathogens.

## Figures and Tables

**Table 1:** Selected peri-implant infections.

Type of Medical Device	Application Example	Most Commonly Isolated Bacterial Strains	Incidence	Reference
Plates and screws for stable plate osteosynthesis	Locomotor system traumatology	*S. aureus, S. epidermidis, S. caprae*	3%–7%	[43,44]
Intramedullary nail	Locomotor system traumatology	*S. aureus, S. epidermidis, S. caprae, E. coli, P. areuginosa*	1%–13%	[45,46,47]
Hip or knee joint prosthesis	Primary arthoplasty	*S. aureus, S. epidermidis, S. caprae, P. mirabilis, P. acnes, P. aeruginosa*	1%–3%	[48,49]

**Table 2:** Chemical composition of Arcam powder.

Composition in wt %							
Alloy	O	V	Al	Fe	H	C	N
Ti6Al4V ELI ASTM F136	<0.13	3.5–4.5	5.5–6.5	<0.25	<0.012	<0.08	<0.05
Ti6Al4V ELI Arcam	0.10	4.0	6.0	0.10	0.010	0.03	0.01
